# A Mixed Micellar Formulation for the Transdermal Delivery of an Indirubin Analog

**DOI:** 10.3390/pharmaceutics12020175

**Published:** 2020-02-19

**Authors:** Seol Hwa Seo, Eunhwan Kim, Yechan Joo, Juseung Lee, Kyung Taek Oh, Sung-Joo Hwang, Kang-Yell Choi

**Affiliations:** 1 Department of Biotechnology, College of Life Science and Biotechnology, Yonsei University, Seoul 03722, Korea; seolhwa87@hotmail.com (S.H.S.); glowlight18@outlook.com (E.K.); 2 College of Pharmacy and Yonsei Institute of Pharmaceutical Sciences, Yonsei University, Seoul 21983, Korea; yechanj@naver.com (Y.J.); ljseung7@gmail.com (J.L.); sjh11@yonsei.ac.kr (S.-J.H.); 3 College of Pharmacy, Chung-ang University, Seoul 06974, Korea; kyungoh@cau.ac.kr; 4 CK Biotechnology Inc., Rm 417, Engineering Research Park, 50 Yonsei Ro, Seodaemun-Gu, Seoul 03722, Korea

**Keywords:** mixed micellar formulation, transdermal drug delivery, solvent-free delivery system, lipophilic drugs

## Abstract

Indirubin is an active component of Dang Gui Long Hui Wan, which has been used in traditional Chinese medicine to treat inflammatory diseases as well as for the prevention and treatment of human cancer, such as chronic myeloid leukemia. The therapeutic effects of indirubin analogs have been underestimated due to its poor water solubility and low bioavailability. To improve the solubility and bioavailability of indirubin analogs, we prepared a mixed micellar formulation with Kolliphor® EL and Tween 80 as surfactants, and PEG 400 as a co-surfactant, followed by complexation with (2-hydroxyproply)-β-cyclodextrin at appropriate ratios. Overall, improving the solubility and skin penetration of indirubin analogs can increase clinical efficacy and provide maximum flux through the skin.

## 1. Introduction

The synthesis of novel compounds based on combinatorial chemistry is important for the improvement of therapeutic activity and industrialization [1]. However, approximately 40% of chemical compounds are limited in their pharmacological activities due to poor water solubility [2]. Although polar organic solvents such as dimethyl sulfoxide (DMSO) have been used for drug solubilization [3], they are limited in their usage in the pharmaceutical market due to biosafety concerns and harmful side effects on human health [4,5].

In recent years, numerous solvent-free delivery systems such as topical ointments, gels, and creams as well as nanocarriers such as nanoparticles, niosomes, liposomes, and mixed micellar formulations were developed to resolve the problem of poorly water-soluble drugs [6,7]. Mixed micelles are fabricated according to the physicochemical properties of the drugs and the compatibility between the micelle core and the drug molecules [7,8,9]. This fabricated micelle structure affords a high solubilization capacity of the bioactive compounds and exhibits a controlled release from the mixed micellar formulation [7]. The development of a mixed micellar formulation provides advantages to a transdermal drug delivery system [10,11]. First, the solubility of both lipophilic and hydrophilic drugs can be increased through microemulsification [10]. Second, microemulsion surfactants, performing as permeation enhancers, can weaken the structure of the stratum corneum (SC) and increase the flux of drugs through the skin [10,12]. Third, drug release from the microemulsion can be enhanced because the affinity of the drug to the internal phase can be easily modified [13]. The choice of an appropriative vehicle for transdermal delivery is critical to improve bioavailability with a minimum dosage of drugs [14]. The most important criteria for the selection of all the mixed micellar formulation components is that all excipients should be pharmaceutically acceptable for oral administration or topical application, depending upon the requirements, and fall under the generally-regarded-as-safe category by the Food and Drug Administration (FDA) [15,16]. Therefore, in this study, we formulated the poorly water-soluble drugs by screening the surfactants and co-surfactants in the safe category, followed by complexation with (2-hydroxyproply)-β-cyclodextrin (HP-β-CD) at 1:1 ratios. HP-β-CD is a family of cyclic oligosaccharides with a hydrophilic outer surface and a lipophilic center cavity [17]. Considering the structure of HP-β-CD, we postulated that the interior cavity of HP-β-CD would encapsulate most of the lipophilic functional groups in KY19382, while the hydrophilic hydroxyl groups at the external surface of the inclusion complex would remain exposed to the environment. HP-β-CD enhances the formulation and stabilization of poorly water-soluble drugs by binding to a drug to form a 1:1 inclusion complex [18]. The formulation was characterized by its ability to form a mixed micellar based on solubility, droplet size, polydispersity, thermodynamic stability, bioavailability, and its safety for topical application. Improving the solubility of indirubin analogs could increase clinical efficacy and could reduce the dosage required to achieve the same effect. Overall, through a series of in vitro and in vivo evaluations, we could successfully improve the solubility and efficiency of poorly water-soluble indirubin analogs by introducing a novel formulation.

## 2. Materials and Methods 

### 2.1. Materials

**Figure 1:**
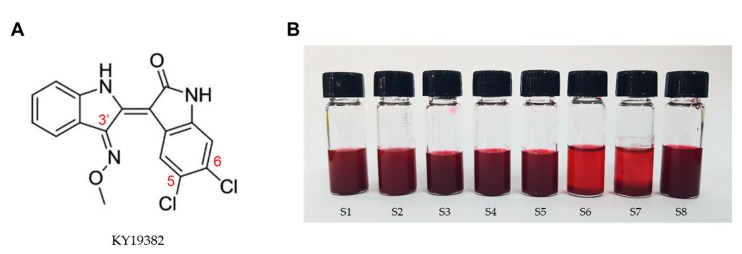
Characterization and solubilization of KY19382. (**A**) Chemical structure of KY19382. (**B**) Solubilization of KY19382 in various vehicles at 25 °C.

KY19382, 5, 6-dichloroindirubin-3′-methoxime (Figure 1a), was synthesized and supplied by Dr. G.H. Han (Yonsei University, Seoul, Korea) [19]. (2-hydroxyproply)-β-cyclodextrin (HP-β-CD), Kolliphor® EL (Polyoxyl castor oil, pH range 6.0–8.0), Tween 20, Tween 80, and polyethylene glycol (PEG) 400 were purchased from Sigma Aldrich (St. Louis, MO, USA). Propylene glycol was purchased from Junsei (Tokyo, Japan). Transcutol P (diethylene glycol monoethyl ether) was purchased from Merck Schuchardt (Hohenbrunn, Germany). All reagents used were analytical grade.

### 2.2. Solubility Test

An excess amount of KY19382 containing HP-β-CD at a 1:1 ratio was tested for its solubility by adding 2 mL of vehicle, independently, in 5 mL-capacity glass vials, followed by mixing for 3 h with a vortex mixer. The mixture samples were centrifuged at 1000× *g* for 10 min to separate the undissolved chemicals. The supernatants were filtered with a 0.45 µm filter membrane, and the concentration of KY19382 was quantified using liquid chromatography–electrospray tandem mass spectrometry (LC-MS) methods. The KY19382 in samples was determined with a LC-MS system (Ultimate 3000 RS-LTQ Orbitrap XL; ThermoFisher Scientific, Waltham, MA, USA) using a C_18_ column (150 × 4.6 mm, 5 µm; ThermoFisher Scientific). The mobile phase contained 60% acetonitrile and 40% distilled water in 0.05% formic acid with a flow rate of 0.7 mL/min at 30 °C. Detection was conducted at a wavelength of 254 nm. The injection volume was 3 µL.

### 2.3. Construction of Phase Diagrams

Surfactant (Tween 80) and co-surfactant (PEG 400) were mixed in different weight ratios (1:2, 1:1, 2:1). For each phase diagram, Kolliphor® EL and specific surfactant/co-surfactant (S_mix)_ ratios were mixed thoroughly in different volume ratios from 1:9 to 9:1 in each glass vial. Pseudoternary phase diagrams were developed using the aqueous titration method. The amount of water added varied in the range of 5% to 95% of the total volume. After each 5% addition of water to the Kolliphor® EL: S_mix_ mixture, transparency and easily flowable condition ratios were recorded. For each water: Kolliphor® EL: S_mix_ ratio, a pseudoternary phase diagram was constructed using CHEMIX School 3.51 software.

### 2.4. Droplet Size and Polydispersity

An aliquot of chosen formulations was diluted with 150 mL water with stirring. The droplet size and zeta potential of the diluted formulations were determined with a zetasizer (ELS-2000ZS; Otsuka Electronics, Osaka, Japan) equipped with a 4.0 mW He-Ne red laser (633 nm) at 25 °C. Experiments were repeated three times. The zetasizer measured the potential, which ranged from −0.12 to 0.12 mV.

### 2.5. Viscosity

The viscosity of the formulations was determined using a Brookfield R/S plus rheometer (Brookfield Engineering, Middleborough, MA, USA) with a C50-1 spindle in triplicate at 25 °C.

### 2.6. Drug Release and Skin Permeation Tests

Drug release and skin permeation tests were performed using Franz Cell diffusion (Logan Instrument Corp., Somerset, NJ, USA). Formulated-indirubin analogs were applied onto human artificial skin (Strat-M™ Membrane Transdermal Diffusion Test Model, Millipore, Billerica, MA, USA) composed of polyethersulfone and polyolefin, then placed into the donor compartment of the Franz cell. The receptor compartment consisted of PBS with 20% methanol. A total of 1 mL of each sample was dropped onto human artificial skin until the formulation covered a diffusion area of 2 × 2 cm^2^. Samples were analyzed at intervals of 0, 1, 2, 3, 4, 5, 6, 8, 10, and 12 h, then quantified using the LC-MS system.

### 2.7. Transmission Electron Microscopy (TEM)

The morphology of micelles was photographed using a transmission electron microscope (Hitachi 7600, Tokyo, Japan) at an accelerating voltage of 100 kV. Briefly, one drop of nanoparticle dispersion was deposited onto a carbon-coated copper grid to form a thin liquid film. The grid was negatively stained immediately by adding a drop of 1% *w*/*w* phosphotungstic acid, followed by air-drying at room temperature for five minutes.

### 2.8. Thermodynamic Stability Study

The sealed formulations were stored at 25 and 4 °C for three months. Then, samples were collected once a month to examine the physical stability of the indirubin analog and emulsifying properties by LC-MS.

### 2.9. Statistical Analysis

Data are presented as the mean ± standard deviation (SD). Statistical analysis was performed using an unpaired two-tailed Student’s *t* test. Asterisks denote statistically significant differences (*, *p* < 0.05; **, *p* < 0.005; ***, *p* < 0.0005).

## 3. Results

### 3.1. Screening of the Mixed Micellar Formulation Components

KY19382, one of the indirubin analogs, had a backbone of 5,6-dichloroindirubin-3′-oxime, which was formed by a π-conjugated system (Figure 1A). To obtain the maximum drug-loading capacity, an excess amount of KY19382 containing HP-β-CD at 1:1 ratio tested the solubility in various oil, surfactant, and co-surfactants (Figure 1B and Figure S1).

The solubility of KY19382 was found to be highest in surfactants compared to oils. The solubility of KY19382 in Kolliphor® EL, Tween 80, and PEG 400 was relatively higher than other excipients (Table 1). Thus, Kolliphor® EL and Tween 80 as surfactants, and PEG 400 as a co-surfactant were selected for the development of the mixed micellar formulation.

### 3.2. Construction of Pseudoternary Phase Diagrams

**Figure 2:**
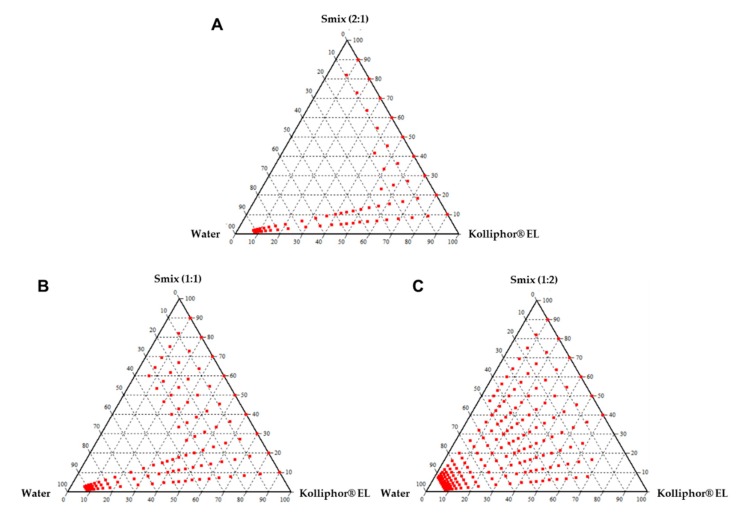
Pseudoternary phase diagrams containing Kolliphor® EL, Tween 80:PEG 400 (S_mix_), and water. (**A**) S_mix_ ratio 2:1, (**B**) S_mix_ ratio 1:1, (**C**) S_mix_ ratio 2:1.

A phase diagram was constructed to identify the soluble regions and to optimize the concentration of the surfactant and co-surfactant in the mixed micellar formulation. A series of formulations at Tween 80/PEG 400 (S_mix_) ratios were prepared at 2:1, 1:1, 1:2 (*v*/*v*). Phase diagrams were constructed of different Kolliphor® EL and S_mix_ ratios from 1:9 to 9:1 (Figure 2A–C). A narrow microemulsion area was observed at the S_mix_ 2:1 (Figure 2A). When the co-surfactant was added with the surfactant in equal amounts, the transparent region increased in area as compared to the region in S_mix_ 2:1 (Figure 2B). On further increasing the co-surfactant ratio, at S_mix_ 1:2, the maximum transparent region was observed (Figure 2C). Hence, the optimal ratio of surfactant to co-surfactant was established to be 1:2 for a three-component mixed micellar formulation.

### 3.3. The Characterization and Evaluation of Mixed Micellar Formulations

We constructed mixed micellar formulations containing increasing concentrations of Kolliphor® EL at a fixed S_mix_ 1:2 ratio (Table 2). Incorporation of the indirubin analog in selective formulations including Kolliphor® EL, Tween 80, and PEG 400 from 50:16:34 (F5), 60:13:27 (F6), 70:10:20 (F7), 80:6:14 (F8), and 90:3:7 (F9) ratios significantly contributed to an increase in the solubility of KY19382 compared to its solubility in Kolliphor® EL only (Table 2). The droplet size decreased with increasing concentrations of Kolliphor® EL in the formulations, whereas viscosity tended to increase with increasing concentrations of Kolliphor® EL (Table 2). We measured the droplet size of the mixed micellar formulation as 41.5 nm (F8) and 29.3 nm (F9), and the zeta potential as −20.38 mV (F8) and −20.99 mV (F9) (Table 2).

**Figure 3:**
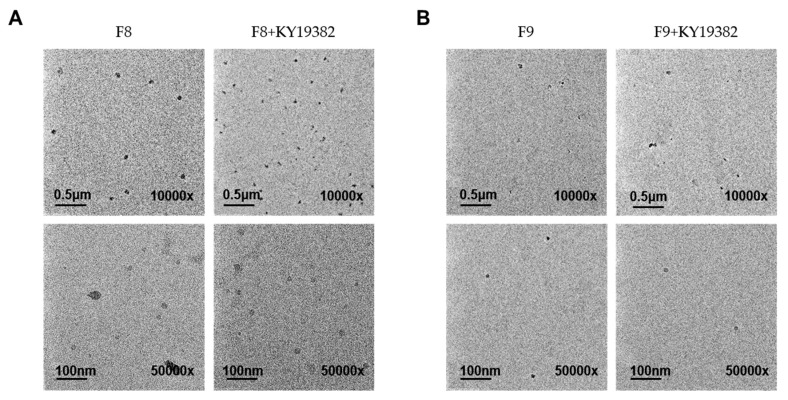
Transmission electronic microscopy (TEM) images of the mixed micellar formation from the (**A**) F8 and (**B**) F9 formulation with or without KY19382. Formulation details are presented in Table 2. The scale bars are 0.5 μm (10,000×) and 100 nm (50,000×), respectively.

TEM images of blank F8, F9, and KY19382-loaded F8, F9 formulations displayed the formation of discrete and monodispersed micelles (Figure 3A,B). The images obtained from F8 and F9 formulations were similar to those prepared with KY19382 (Figure 3A,B). Furthermore, drug loading did not influence the size of the nanoparticles, and showed no apparent signs of aggregation (Figure 3A,B).

Therefore, the selected F8 and F9 formulations were appropriated to the mixed micellar formulation with a particle size of <100 nm in the stability of the mixed micellar formulation. Based on the above results, the mixed micellar formulations of F8 and F9 possessed the optimal emulsifying properties among all examined formulations. In addition, the mixed micellar formulations F8 and F9 may increase skin permeability due to the small droplet size, and may be more convenient for administration onto the skin due to their high viscosity.

### 3.4. Skin Permeation Study

**Figure 4:**
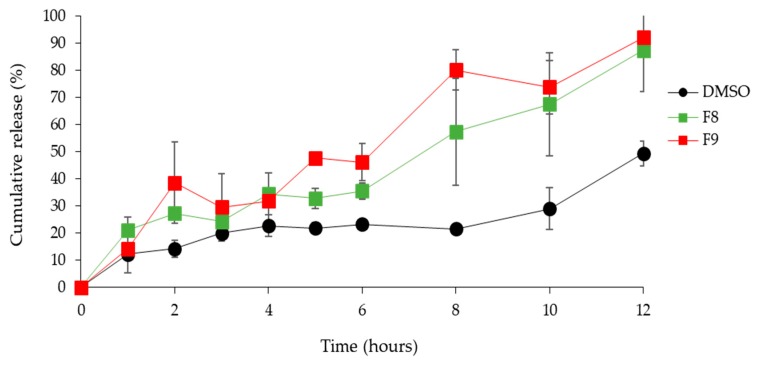
KY19382 transdermal permeation profiles obtained after application of different mixed micellar formations or KY19382 in DMSO to artificial skin. Formulation details are presented in Table 2. Data are presented as the mean ± standard deviation (SD).

Skin permeation studies showed a significant increase in the KY19382 permeation in the F8 and F9 formulations compared with that of the permeation in DMSO (Figure 4), indicating that the transdermal delivery of KY19382 from the mixed micellar formulation played a key role in the enhancement of both the penetration and release rate.

### 3.5. Thermodynamic Stability of the Mixed Micellar Formulations

**Figure 5:**
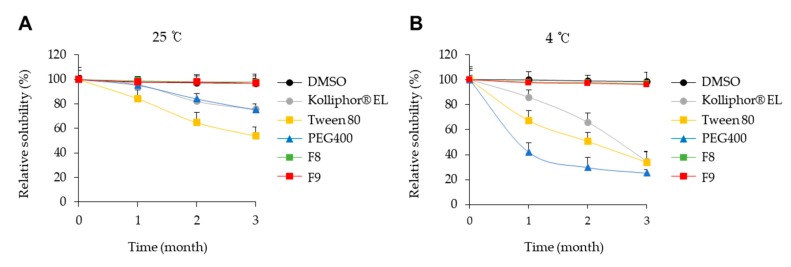
Time evolution of the concentration of KY19382 in F8 and F9 formulations. Concentrations of KY19382 at 25 °C (**A**) and 4 °C (**B**).

To confer a long shelf-life to the mixed micellar formulation, as compared to ordinary emulsion, aliquots of KY19382 in DMSO, Kolliphor® EL, Tween 80, PEG 400, and mixed micellar formulation (F8, F9) were stored at 25 °C and 4 °C for three months. KY19382 was thermodynamically stable in the optimized mixed micellar formulation; therefore, it maintained physical stability without precipitation for up to three months of storage at 4 °C and 25 °C (Figure 5A,B). However, the stability of KY19382 was significantly reduced in Kolliphor® EL, Tween 80, and PEG 400 after storage at 4 °C (Figure 5B). KY19382 formulated with individual excipients such as Kolliphor® EL, Tween 80 and PEG 400 was unstable, and the drug solution had a tendency to return to its equilibrium state via precipitation (Figure 5A,B). To test whether these results were due to chemical interactions between KY19382 and excipients, we assessed the chemical stability of the formulations. The degradation products were not monitored in a validated HPLC assay (Figure S2). Altogether, these findings indicated that the mixed micellar formulations, including Kolliphor® EL, Tween 80 and PEG 400, had thermodynamic stability in comparison with formulations made with the individual excipients.

## 4. Discussion

Here, we formulated a mixed micellar based delivery system to improve the solubility of the poorly water-soluble indirubin analog. Indirubin analogs have one hexameric ring formed by intramolecular hydrogen bonds [20,21]. Therefore, the KY19382 molecule was stable and poorly water-soluble.

There are various systems, including solid dispersion, liposomes, polymer micelles, nanoemulsions, and cyclodextrin that are developed for the enhancement of drug solubility [22,23]. Considering the molecular characteristics of KY19382, we selected HP-β-CD as a solubility enhancer from a list of FDA-approved substances. However, HP-β-CD is limited in its penetration into lipophilic membranes [24]. To overcome this bioavailability problem, we optimized a mixed micellar formulation composed of HP-β-CD with various oils, surfactants, and co-surfactants. The PEG 400 molecule opens two parallel helical bonds [25] so that the indirubin molecule complex and HP-β-CD are incorporated into the coiling chain and form a molecular dispersion. Meanwhile, the intramolecular hydrogen bond was activated upon dissolving the indirubin analog in a mixed micellar formulation, so exchange of a hydrogen bond with PEG 400 may occur [26]. Tween 80, which is derived from oleic acid, contributes to the fluidity increments of the SC lipids, enhancing the permeability of the skin for drugs [27,28]. In addition, the presence of monooleate, such as Kolliphor® EL, in the formulation further improves bioavailability by affecting the drug absorption in the skin [29].

A mixed micellar formulation is a function of system composition [30,31]. The optimal ratio of the surfactant and co-surfactant is a major determining factor that influences the phase properties, droplet size, and position of the microemulsion region [30,31]. When Tween 80 was added with PEG 400 in the 1:2 ratio, a higher transparent region was observed as compared to the 1:1 and 2:1 ratios, perhaps due to the further reduction in the interfacial tension and increased fluidity of the interface at S_mix_ 1:2. It was also observed that increasing the Kolliphor® EL ratio led to an increased region of mixed micellar formulation. The concentration of Kolliphor® EL is a major factor influencing the phase properties, droplet size, and position of the liquid crystalline region [32]. The high loading capacity of the formulation components can be attributed to the unique structural organization of the microemulsion system [33,34]. We confirmed that the droplet size of mixed micellar formulation was 41.5 nm (F8) and 29.3 nm (F9), and the zeta potential as −20.38 mV (F8) and −20.99 mV (F9). In general, the zeta potential value of ±30 mV is sufficient for the stability of a microemulsion [35].

The transdermal drug release rate is correlated with the flux values; therefore, a smaller droplet dimension and lower interfacial tension of the mixed micellar formulation is assumed to improved drug permeation [36,37]. Higher surfactant concentration causes an increase in the fluidity of the membrane, and eventually an increase in the permeability of the drug [38]. Therefore, it has been suggested that a dose-dependent increase in the permeability of the SC is responsible for the surfactant-induced permeability of drugs.

Mixed micellar formulations also present an advantage in storage stability [7]. KY19382 formulated with individual excipients such as Kolliphor® EL, Tween 80, and PEG 400 was unstable, and the drug solution had a tendency to return to its equilibrium state via precipitation after 3 months of storage at 4 °C and 25 °C. Due to this instability, additional stabilization was required by mixed micellar formulation. After KY19382 was formulated, it showed thermodynamic stability and precipitation was not observed in the optimized formulation after 3 months of storage at 4 and 25 °C.

In conclusion, based on a series of in vitro and in vivo evaluations, we demonstrated a mixed micellar formulation which is an effective approach for the dissolution of poorly water-soluble drugs. A mixed micellar formulation promotes bioavailability and permeability across cellular lipid membranes with smaller droplets. Although further detailed examinations are required, the findings of this study suggest that developing a mixed micellar formulation is a promising method to enhance transdermal drug delivery while reducing the potential risk of side effects.

## 5. Conclusions

A mixed micellar formulation is a promising approach for the enhancement of transdermal drug delivery. In the present study, we developed a mixed micellar formulation that included Kolliphor® EL, Tween 80, and PEG 400 for efficient administration of poorly water-soluble compounds by an enhancement of their solubility and bioavailability. The mixed micellar formulation could provide the excipients with high flexibility at the optimum viscosity, as the formulations retained their efficacies after phase transition. In addition, the mixed micellar formulation enhanced transdermal delivery with a nano-sized droplet. All the excipients for the mixed micellar formulation were pharmaceutically acceptable for topical application based on the safety guidelines of the FDA. Therefore, the mixed micellar formulation developed in this study could be a promising approach for human dermal delivery of poorly water-soluble drugs.

## Figures and Tables

**Table 1:** Solubility of KY19382 in selected excipients.

Function in Formulation		Vehicles	Solubility (mg/mL)	*p*-Value
Oils	S1	Olive oil	0.83 ± 0.02	0.0312
	S2	Sunflower oil	1.35 ± 0.98	0.0182
Surfactants	S3	Kolliphor® EL	5.4 ± 0.95	0.0031
	S4	Tween 20	4.58 ± 0.13	0.0269
	S5	Tween 80	4.92 ± 0.23	0.0005
Co-surfactants	S6	Transcutol P	1.15 ± 0.87	0.0443
	S7	Propylene glycol	0.25 ± 0.03	0.0052
	S8	PEG 400	3.1 ± 0.46	0.0003

**Table 2:** Solubility, droplet size, viscosity, and zetapotential for the mixed micellar formulations.

Code	Formulation Composition (Kolliphor® EL:Tween 80:PEG400)	Solubility (mg/mL)	Droplet size (nm)	Viscosity (mPas)	Zetapotential (mV)
F1	10:30:60	2.99 ± 0.20	1525.7 ± 10.6	23.33 ± 0.85	−5.14 ± 0.14
F2	20:26:54	3.58 ± 0.13	921.3 ± 2.6	44.46 ± 1.35	−6.87 ± 0.15
F3	30:23:47	3.81 ± 0.04	624.9 ± 9.2	59.12 ± 1.66	−7.23 ± 0.17
F4	40:20:40	4.11 ± 0.20	479.4 ± 3.8	65.07 ± 1.35	−6.94 ± 0.16
F5	50:16:34	5.67 ± 0.23	332.3 ± 3.8	72.72 ± 1.17	−7.99 ± 0.18
F6	60:13:27	6.86 ± 0.12	264.9 ± 4.2	88.85 ± 0.98	−9.72 ± 0.20
F7	70:10:20	7.59 ± 0.23	183.8 ± 2.2	91.39 ± 0.56	−10.83 ± 0.22
F8	80:6:14	7.69 ± 0.17	41.5 ± 1.7	98.3 ± 0.91	−20.38 ± 0.32
F9	90:3:7	6.88 ± 0.07	29.3 ± 1.0	99.52 ± 1.27	−20.99 ± 0.33

## Supplementary Materials

The following are available online at https://www.mdpi.com/1999-4923/12/2/175/s1, Figure S1: Schematic representation of the mechanism of a mixed micellar formulations, Figure S2: HPLC chromatograms of KY19382 in DMSO, Kolliphor® EL, Tween 80, and PEG400.
