# Supplementary material for: A Mixed Micellar Formulation for the Transdermal Delivery of an Indirubin Analog
Source: Pharmaceutics. 2020 Feb 19;12(2):175. doi: 10.3390/pharmaceutics12020175 (PMC7076637; doi:10.3390/pharmaceutics12020175)
Supplement: Supplementary file 1 [file pharmaceutics-12-00175-s001.pdf]

pharmaceutics

Supplementary Materials: A mixed micellar formulation for the transdermal delivery of an indirubin analog

Seol Hwa Seo, Eunhwan Kim, Yechan Joo, Juseung Lee, Kyung Taek Oh, Sung-Joo Hwang, and Kang-Yell Choi

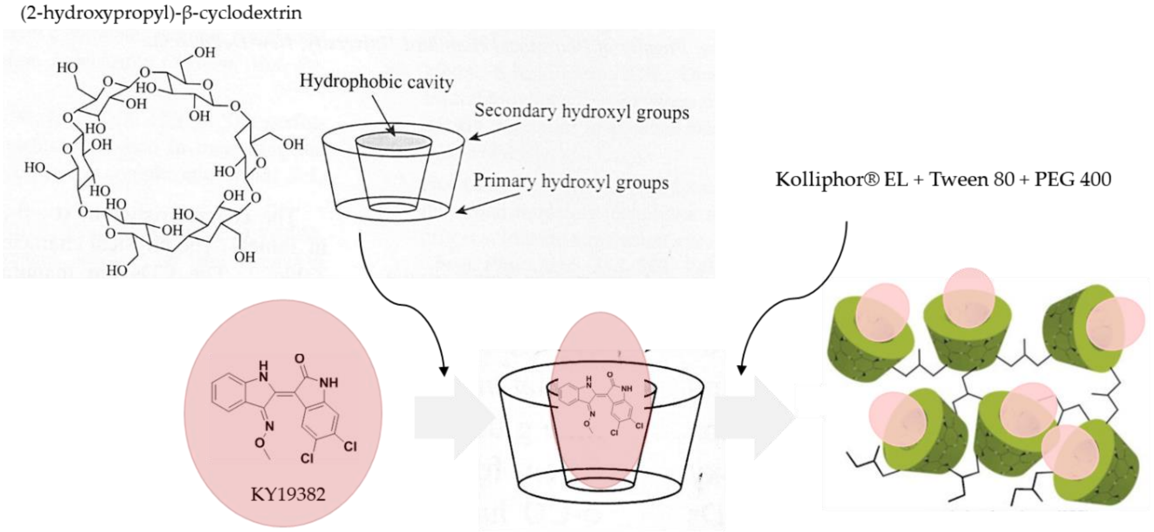

Figure S1. Schematic representation of the mechanism of a mixed micellar formulations. KY19382 mixed with (2-hydroxypropyl)-β-cyclodextrin (HP-β-CD) which encapsulates most of the lipophilic functional groups in KY19382 while the hydrophilic hydroxyl groups at the external surface of the inclusion complex. The indirubin molecule complex with HP-β-CD are incorporated into the coiling chain with Kolliphor® EL, Tween 80, and PEG 400, and formed a molecular dispersion.

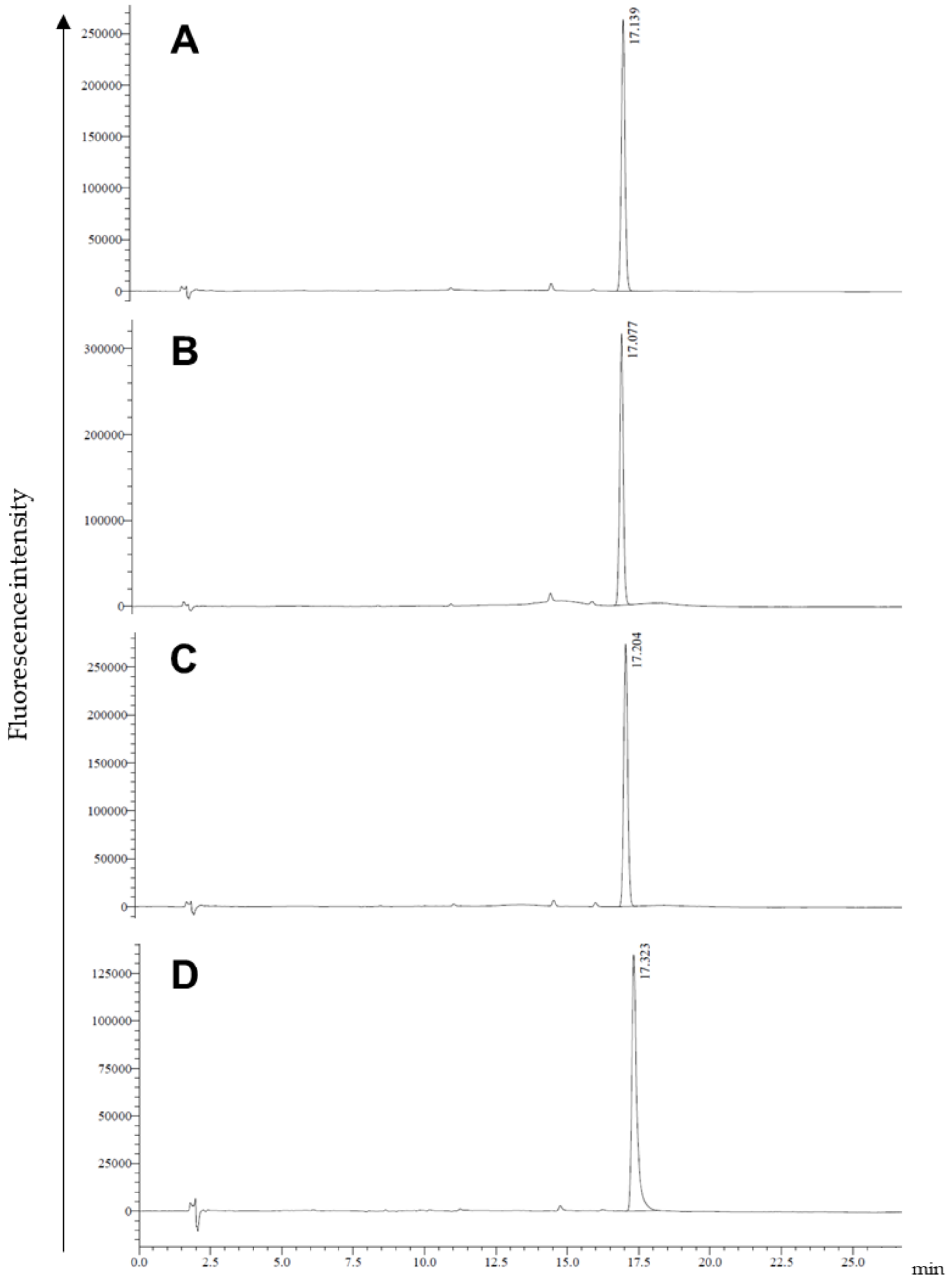

Figure S2. HPLC chromatograms of KY19382 in DMSO (A), Kolliphor® EL (B), Tween 80 (C), and PEG400 (D). All chromatograms were obtained by injection of equal volumes of micellar formulation from the same quantity of KY19382. The numbered peaks were determined by MS and MS/MS.
